# Affinity2Vec: drug-target binding affinity prediction through representation learning, graph mining, and machine learning

**DOI:** 10.1038/s41598-022-08787-9

**Published:** 2022-03-19

**Authors:** Maha A. Thafar, Mona Alshahrani, Somayah Albaradei, Takashi Gojobori, Magbubah Essack, Xin Gao

**Affiliations:** 1 Computer, Electrical and Mathematical Sciences and Engineering (CEMSE) Division, Computational Bioscience Research Center (CBRC), King Abdullah University of Science and Technology (KAUST), Thuwal, Saudi Arabia; 2 College of Computers and Information Technology, Taif University, Taif, Saudi Arabia; 3 National Center for Artificial Intelligence (NCAI), Saudi Data and Artificial Intelligence Authority (SDAIA), Riyadh, Saudi Arabia; 4 Faculty of Computing and Information Technology, King Abdulaziz University, Jeddah, Saudi Arabia

**Keywords:** Computational models, Data integration, Machine learning

## Abstract

Drug-target interaction (DTI) prediction plays a crucial role in drug repositioning and virtual drug screening. Most DTI prediction methods cast the problem as a binary classification task to predict if interactions exist or as a regression task to predict continuous values that indicate a drug's ability to bind to a specific target. The regression-based methods provide insight beyond the binary relationship. However, most of these methods require the three-dimensional (3D) structural information of targets which are still not generally available to the targets. Despite this bottleneck, only a few methods address the drug-target binding affinity (DTBA) problem from a non-structure-based approach to avoid the 3D structure limitations. Here we propose Affinity2Vec, as a novel regression-based method that formulates the entire task as a graph-based problem. To develop this method, we constructed a weighted heterogeneous graph that integrates data from several sources, including drug-drug similarity, target-target similarity, and drug-target binding affinities. Affinity2Vec further combines several computational techniques from feature representation learning, graph mining, and machine learning to generate or extract features, build the model, and predict the binding affinity between the drug and the target with no 3D structural data. We conducted extensive experiments to evaluate and demonstrate the robustness and efficiency of the proposed method on benchmark datasets used in state-of-the-art non-structured-based drug-target binding affinity studies. Affinity2Vec showed superior and competitive results compared to the state-of-the-art methods based on several evaluation metrics, including mean squared error, rm2, concordance index, and area under the precision-recall curve.

## Introduction

Drug repositioning has gained significant attention due to its reduced time, lower investment, and higher success rate compared to the traditional de novo drug development^1^. This realization and large repositories of drug-related data being made accessible to researchers spurred on the development of a number of computational DR strategies^2^. One of the significant strategies is computational drug-target interaction (DTI) prediction, as it narrows down the search space for candidate drugs that can treat targeted diseases^3^. DTI prediction methods can be classified into two main categories based on the prediction task. The first is the binary classification category in which the prediction task is to determine if the drug interacts with the target protein. The second is the regression category in which the prediction task is to determine continuous values that indicate the strength of the binding between the drug and the target (i.e., the binding affinity)^4^.

The more commonly developed binary classification methods range from docking- and chemogenomic-based approaches^5–7^ to network-based^8,9^ and machine learning (ML)-based approaches^10–14^. The network-based approaches include knowledge graph-based^14^ and graph embedding-based approaches^15,16^. These prediction types cannot differentiate the actual negative DTIs and interactions with missing information (unknown values), negatively affecting the model prediction performance. Moreover, the prediction does not reflect the binding strength between the drugs and proteins that impact the drug's potential efficacy. Thus, more current research focuses on drug-target binding affinity (DTBA) prediction that builds regression models instead of classification models. Developing a regression-based method can rank the therapeutic drugs, further limiting the scope of potential drugs (i.e., compounds) for drug discovery studies. Moreover, these DTBA regression models overcome previous limitations by having the ability to reflect the binding strength through predicting the affinity values using different measurements such as inhibition constant (K_i_), dissociation constant (K_d_), or the half-maximal inhibitory concentration (IC_50_)^17^. However, several DTBA prediction methods developed to date require the three-dimensional (3D) structure of drugs or target proteins which are mostly not available and cannot be scaled to large-scale data due to the low quality of 3D structures^18,19^. Moreover, these structure-based methods required a molecular docking step, which is a bottleneck associated with prediction efficiency.

The more recently developed ML and deep learning (DL)-based methods avoid this limitation by using non-structure-based methods (i.e., sequence-based methods) that do not require docking or 3D structural data for DTBA predictions^4^. Also, these ML/DL-based methods can aid and support the other types of methods in affinity prediction. Despite those methods' impressive performance, the DTBA regression task remains a critical and challenging task; there is more room to develop several algorithms that improve the prediction performance. The first attempt to predict DTBA without using 3D structural data is Kronecker-Regularized Least Squares (KronRLS)^20^. KronRLS is an ML-based method that uses target-target similarity and drug-drug similarity matrices as features to predict DTBA values by finding a minimizer of the objective function (i.e., error or loss function). The second ML method, SimBoost^21^, uses similarity matrices and constructed features obtained from target-target similarity, drug-drug similarity, and drug-target networks to predict DTBA using gradient boosting regression trees. SimBoost improved the prediction performance compared to the KronRLS method. However, more recently, with the similarity-based DL method, SimCNN-DTA^22^ achieved even better prediction performance. SimCNN-DTA predicts DTBA values by applying a two-dimensional convolutional neural network (CNN) on the outer product of both the target similarity and drug similarity matrices. DL-based DTBA methods that are not similarity-based have also been developed^23–27^. DeepDTA^23^ is one of the first DTBA prediction methods developed by constructing two CNNs applied on drug SMILES and target amino-acid sequences to learn feature representations for drugs and targets, respectively. These feature representations were subsequently combined and fed to fully connected layers for DTBA prediction. Similar to DeepDTA, several DL-based methods have been established using similar concepts but different in: using other SMILES and sequences embeddings^26^, integrating different input representations such as gene-ontology for proteins^28^ or molecular graphs for drugs^29^, using different neural networks (NN) architectures for feature representation learning^30^, or utilizing different DL techniques such as long-short-term memory (LSTM) or attention mechanisms^27^.

Several Network-based methods^31–33^ outperform other DTI prediction approaches. For example, AOPEDF^34^ created a heterogeneous biological network by integrating drugs, proteins, and diseases. Then AOPEDF learns a low-dimensional vector representation of features that preserve arbitrary-order proximity from this rich constructed network covering chemical, genomic, phenotypic, and network profiles. Finally, a cascade deep forest classifier was built to identify novel DTIs. Another network-based approach, MultiDTI^35^, uses a joint representation framework (i.e., multi-modal representation) based on heterogeneous networks to combine similarity-based methods with network-based methods. MultiDTI takes advantage of the comprehensive information and different perspectives for DTI prediction. First, MultiDTI combined the interaction/association information of the heterogeneous network and the drug/target sequence information and then mapped the drugs, targets, side effects, and disease nodes in the heterogeneous network into a shared space. Finally, it predicted new DTIs based on the distance between the drug and the target in this new shared space. The last but very recent network-based DTI prediction method, DTi2Vec^36^, integrates the drug-drug similarity-graph, the target-target similarity graph, and DTIs into one weighted heterogeneous graph. After that, it applied graph embeddings on this network to generate node embeddings and then learned feature representation for each edge (drug-target pair) used to predict DTIs. These three methods are formulated as supervised link prediction problems (i.e., binary classification) and proved their efficiency in performance evaluation in terms of AUPR and AUC by outperforming several state-of-the-art methods and predicting novel DTIs. Similar to these methods, in our work, we here leverage the power of network characteristics combined with other techniques to predict binding affinities for drug-target pairs. Thus, we constructed a heterogeneous network by integrating several networks, but we did not include any disease-related network data. However, our method objective is to predict binding affinities (bioactivity continuous values) for drug-target pairs instead of on/off binary relationships and use the appropriate evaluation metrics.

This study is the first regression-based attempt to deal with the drug-target prediction task as a whole weighted heterogeneous network to the best of our knowledge. We constructed a weighted network using three graphs (i.e., binding affinity graph, target-target similarity graph, and drug-drug similarity graph) and calculated the similarities using several techniques. Here we present Affinity2Vec, as a novel network-based method that accurately predicts the continuous binding affinity values between drugs (i.e., compounds or ligands) and target proteins (more specifically, kinases). Affinity2Vec integrates several techniques, including amino-acid sequence embeddings, SMILES embeddings, graph mining for feature extraction, and ML for prediction. Our method outperforms the baseline methods using the benchmark datasets based on multiple evaluation metrics and notably reduced prediction errors.

## Materials

### Benchmark datasets

Two biomedical datasets specialized for DTBA prediction, Davis^37^ and KIBA^38^ datasets, have become benchmark datasets to train and evaluate non-structure-based methods. Table 1 provides a summary of those two datasets. Davis dataset^37^ has selectivity assay data for the Kinase proteins family. It consists of 68 unique drugs, 442 unique target proteins, and 30,056 binding affinity values for all the drug-target pairs. The binding affinity between the drug-target pair is measured by kinase dissociation constant (K_d_). The higher the value of K_d_, the lower binding between drug and target. Similar to the DL-baseline methods^23^, the affinity values were transformed into logarithm space (pK_d_) by applying the following equation:

**Table 1:** Statistics for the drug-target binding affinity benchmark datasets.

Datasets	No. of drugs	No. of proteins	Known DTBA	Density (%)	Refs
Davis	68	442	30,056	100	^37^
KIBA	2116	229	118,254	24.4	^38^

1 $${pK}_{d} = -{\text{log}}_{10}(\frac{{K}_{d}}{1e9})$$

The affinity values in the Davis dataset are ranging from 5.0 to 10.8. KIBA dataset^38^ provides Kinase Inhibitor BioActivity (KIBA) data, and it introduces KIBA scores that combine the information from different sources, including K_d_, K_i_ (inhibitor constant), and IC_50_ (the concentration required to produce half-maximum inhibition) into a single bioactivity score. Following prior work^39^, we filtered the KIBA dataset by keeping the drugs and proteins with 10 known interactions or more, resulting in 2,111 drugs and 229 targets with 118,254 interactions. KIBA affinity scores are ranged from 0.0 to 17.2. The lower the KIBA score the stronger the binding affinity between the drug and the target. The density indicates the percentage of known binding affinities.

In addition to these two datasets, we included a dataset recently used for protein–ligand complexes binding affinity prediction, named the PDBBind dataset^40^, as a third benchmark dataset to test and assess our method. PDBBind dataset is a comprehensive resource of experimentally measured binding affinity data expressed with pKd values for protein–ligand complexes provided with the 3D structural information of molecular complexes in the form of SDF, PDB, and MOL2 files. PDBBind datasets are derived from the Protein Data Bank (PDB)^40^ and consist of three subsets: the general set, the refined set, and the core set. The general set contains lower quality information of the complexes, and the refined set is a subset of the general set with better quality information (i.e., high structural resolution, accurate binding measurements, and the nature of the complexes). The core set is the highest quality benchmark subset. However, its size is very small compared to the other subsets. Therefore, we used the widely used PDBBind Refined v.2015 dataset to take advantage of the high-quality data with a reasonable size. We utilized PDBbind Refined dataset v.2015 with 3437 protein–ligand complexes with their affinity values similar to previously published works^41–43^. Briefly, we filtered this number to 3045 pairs for which we managed to obtain amino-acid sequences for the proteins and SMILES for the ligands.

### Input representation

#### Drug SMILES

Our study represents the drugs using the Simplified Molecular-Input Line-Entry System (SMILES)^44^, a line notation that describes chemical compounds' structure as text strings. We collected the drugs’ SMILES for both Davis and KIBA datasets from the previous work^23^. For drugs in the Davis dataset, they were extracted from the PubChem compound database based on their PubChem CIDs^45^. For drugs in the KIBA dataset, they used the PubChem CIDs to extract the SMILES. For the Davis datasets’ drugs, the maximum and average lengths of SMILES are 103 and 64 tokens, respectively. While for the KIBA datasets’ drugs, the maximum and average lengths of SMILES are 590 and 58 tokens, respectively. For the PDBbind Refined dataset, we first obtained SDF files for all ligands from the PDBBind webserver for v.2015. After that, we converted these SDF files to SMILES using RDKit^46^ with python application programming interface (API), an open-source Cheminformatics software. For this dataset, the maximum and the average length of SMILES are 126 and 46 tokens, respectively.

#### Protein sequences

We also acquired the proteins' amino-acid sequences from previous work by^23^. They extracted protein sequences from the UniProt protein database using gene names/RefSeq accession numbers for the Davis dataset and UniProt IDs for the KIBA dataset^47^. The maximum and average length of protein sequences are 2549 and 788 characters for the Davis dataset, respectively. While the maximum and average length of protein sequences are 4128 and 728 characters for the KIBA dataset, respectively. For the PDBbind Refined dataset, we retrieved the Fasta file for all the proteins from the prior work^42^, initially extracted from the PDBBind database server. The maximum and average length of protein sequences are 4638 and 494 characters, respectively.

#### Drug and target similarity

We retrieved drug similarity scores for all drug pairs and target similarity scores for all target pairs for both Davis and KIBA datasets from previous works in an adjacency matrix format^23,39^. They calculated the drug similarity scores using the SIMCOMP tool^48^ that represented the drugs by their two-dimensional (2D) chemical structure (as a graph). Then, calculated the drug-drug similarity score based on the common sub-structures size of the two graphs using the Tanimoto coefficient. For target similarity scores, they represented the targets using amino-acid sequences. They then calculated the target similarity scores using the normalized Smith-Waterman (SW) scores^49^ based on the protein sequences' alignment. We refer to the drug similarity matrix and the target similarity matrix as *DDsim1* and *TTsim1*, respectively, for later use.

## Methods

### Problem formulation

This work presents a regression-based approach for predicting binding affinity scores. The input data consists of: *D* = *{d*_*1*_*, d*_*2*_*, …, d*_*n*_*}* where D represents the drug space, and the number of drugs is n, *T* = *{t*_*1*_*, t*_*2*_*, …, t*_*m*_*}* where T represent the target protein space, and the number of target proteins is m, and finally *Y* = *{y*_*ij*_^*N*^*}* which is the label (i.e., continuous values of binding affinity) where *y*_*ij*_ is the binding scores between drug_i_-target_j_ pair and N is the number of observed affinities. For each drug-target pair, we extracted different features using several techniques, explained later in the feature extraction section. The feature vector (FV) is represented by *X ⊆ {x*_*1*_*, x*_*2*_*, …, x*_n*m_*}* and their labels (i.e., continuous values) *Y ⊆ {y*_*1*_*, y*_*2*_*, …, y*_n*m_*}* where n*m is the number of all possible (drug, target) pairs. It is necessary to mention that, in the Davis dataset, all drug-target pairs have affinity values (i.e., labels), while in the KIBA and PDBBind Refined datasets, many pairs have ‘NaN’ labels which we excluded from *X* and *Y*. Most of the previous works generated each drug-target pairs’ features using different techniques applied on SMILES and sequences of drugs and proteins, respectively. However, up to now, there is no published work that deals with binding affinity prediction problems as a network-based problem which we adjust in our study.

### Overview of the Affinity2Vec model

**Figure 1:**
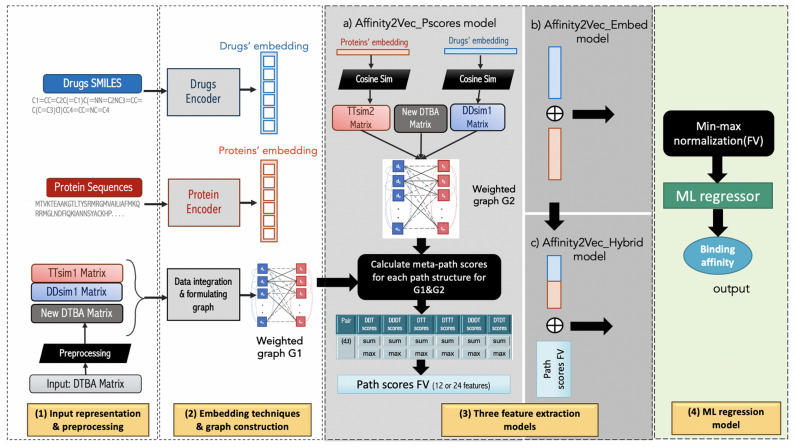
The pipeline of Affinity2Vec, which consists of four main steps and three models for feature extraction a, b, and c.

Figure 1 illustrates the pipeline for Affinity2Vec, which consists of four main parts:

1. Input representations, data preprocessing, and constructing heterogeneous network for the DTBA.
2. Applying embedding techniques on the drugs’ SMILES and targets’ sequences to generate feature representations for individual drugs and target proteins. Also, we integrated the binding affinity, drug similarity, and target similarity graphs to form the complete DTBA network.
3. Applying three feature extraction techniques to generate three different feature types: the embedding features, the meta-path score features, and the hybrid model that combine the previous two types of features.
4. Applying ML regressors to establish the regression models.

We explain each of these steps in more details in the corresponding sections.

### Preprocessing and constructing DTBA network

In our study, we constructed a weighted heterogeneous graph *G(V, E)* using DTBA network integrated with target-target similarity and drug-drug similarity graphs, where *V* is the set of vertices (i.e., drug and target nodes) and *E* is the set of edges that represent one of three types of edge: (1) *D-T* edges reflect the binding strength values between drugs and targets, (2) *D-D* edges or *T-T* edges represent the connection between two drugs or two target proteins, respectively, with a similarity score above a certain threshold. It is essential to mention that we used two similarity matrices for drugs and targets, (*DDsim1, TTsim1*) and (*DDsim2, TTsim2*), from different sources to construct heterogeneous graphs *G1* and *G2*, respectively, which are used later in graph-based feature extraction. We explain the formulation of the graph edges in more details in subsections that follow.

#### Filtering similarity edges

First, we normalized each similarity graph separately to have the similarity scores ranging within [0,1]. Before integrating the target-target similarity and drug-drug similarity graphs, we applied a preprocessing step by filtering each graph separately. If we include all similarity scores as edges connecting similar target proteins or similar drugs, the graph will be a very complex network, specifically when the number of targets or drugs is large. Therefore, we removed all the weak similarity scores for the target similarity graph by filtering the edges within a specific threshold. We applied the same process to filter the drug similarity graph. We did an empirical analysis for the target-target similarity and drug-drug similarity graphs separately, as explained and illustrated in the Supplementary Material, Fig. S1, to specify different threshold values for each graph. For the Davis dataset as an example, the threshold for the drugs 2D chemical structure similarity is set to 0.3 while the threshold of the targets normalized Smith-Waterman alignment similarity is set to 0.04. Thus, each similarity edge below the threshold is removed, resulting in getting target-target similarity and drug-drug similarity subgraphs. The insight of applying this process is to reduce the noisy information introduced when using all similarity scores, including those shallow similarity scores, which do not provide any informative meaning that affects the performance. That is, decreasing the edges in each similarity graph reduces the model’s running time.

#### Formulating binding affinity edges

In Davis, KIBA, and PDBBind datasets, the lower affinity values indicate the stronger binding between the drug and the target. Thus, the binding affinity values have a similar meaning of distance (e.g., the smaller distance between the two entities, the stronger relations between them), which has the opposite meaning of the similarity edge used in the target-target similarity and drug-drug similarity graphs. As a result, we need to have a consistent meaning for all edge types (the higher value of edge, the stronger relationship) and a consistent value range (i.e., [0,1]) in this heterogeneous network. For this purpose, applying an exponential function is a potentially good candidate to convert the affinity values (the element which has a high value should be converted into a low value) and output them in the range [0,1]. Another function that fits our goal is a SoftMax function, a form of logistic regression (i.e., normalized exponential function), which normalizes the input value into a value vector that follows a probability distribution whose total sums up to one. We applied both functions on the binding affinity values for the three datasets separately and adopted the best result approach. The exponential and SoftMax $\sigma$ functions^50^ are described in Eqs. (2) and (3), respectively.

2 $$f({z)}_{i}= {e}^{(-\alpha {z}_{i})}$$

3 $$\sigma ({z)}_{i} =\frac{{e}^{{z}_{i}}}{{\sum }_{j=1}^{K}{e}^{{z}_{j}}} ,\text{for i}=1, ...,\text{ K and }z=({z}_{1}, \dots , {z}_{k}) \in {\mathbb{R}}^{K}$$

where *z* is the data samples of affinity values, and parameter alpha $\alpha$ in Eq. (2) can be tuned or possibly estimated from the data. After preprocessing the affinity values, we augmented the DTBA with new affinity values, and target-target similarity and drug-drug similarity subgraphs to construct the extensive DTBA heterogeneous network, which is used later in the graph-based feature extraction step.

### Learning representation for drugs and proteins

In this step, the inputs are the SMILES of the drugs and the amino-acid sequences for the target proteins, and then different techniques are applied to generate embeddings for each drug and each target protein.

### Drug encoders

To capture the critical properties and generate features for each drug, we applied the sequence-to-sequence learning model (seq2seq, aka encoder-decoder model)^51^. Specifically, we adopted the seq2seq fingerprint model introduced in^52^. This unsupervised data-driven DL molecular embedding method applied several modifications into the original version of the seq2seq learning model to fit drug discovery tasks that are in our scope. Our objective is to learn the vital features of the drugs. We accommodated and adjusted this version of the seq2seq model to encode drugs that suit our work for the following reasons: (1) this method is data-driven, and there is no need for any expert knowledge; (2) the fingerprints (i.e., feature representations) generated by this method can construct the original drug representations, which means it ensures that the information encoded in the fingerprint vector is sufficient; (3) this method uses unsupervised training on a substantial unlabeled dataset by applying the power of deep neural networks (DNN). The seq2seq model that we applied consists of a multi-layered Gated Recurrent Unit (GRU) network that maps the input into a fixed dimension FV, and a deep GRU network that decodes the FV back to the original form. It is necessary to mention that the seq2seq model implemented GRU instead of long short-term memory (LSTM), usually implemented in the encoder-decoder model, which has similar performance but it accelerates the training process. A dropout layer is attached to avoid overfitting in the training phase. The last thing to mention, an extra fingerprint extraction layer set is fed-forwarded to only the perceiver network to extract the resulting fingerprint embeddings, which we take advantage of to obtain the embeddings for each drug.

**Figure 2:**
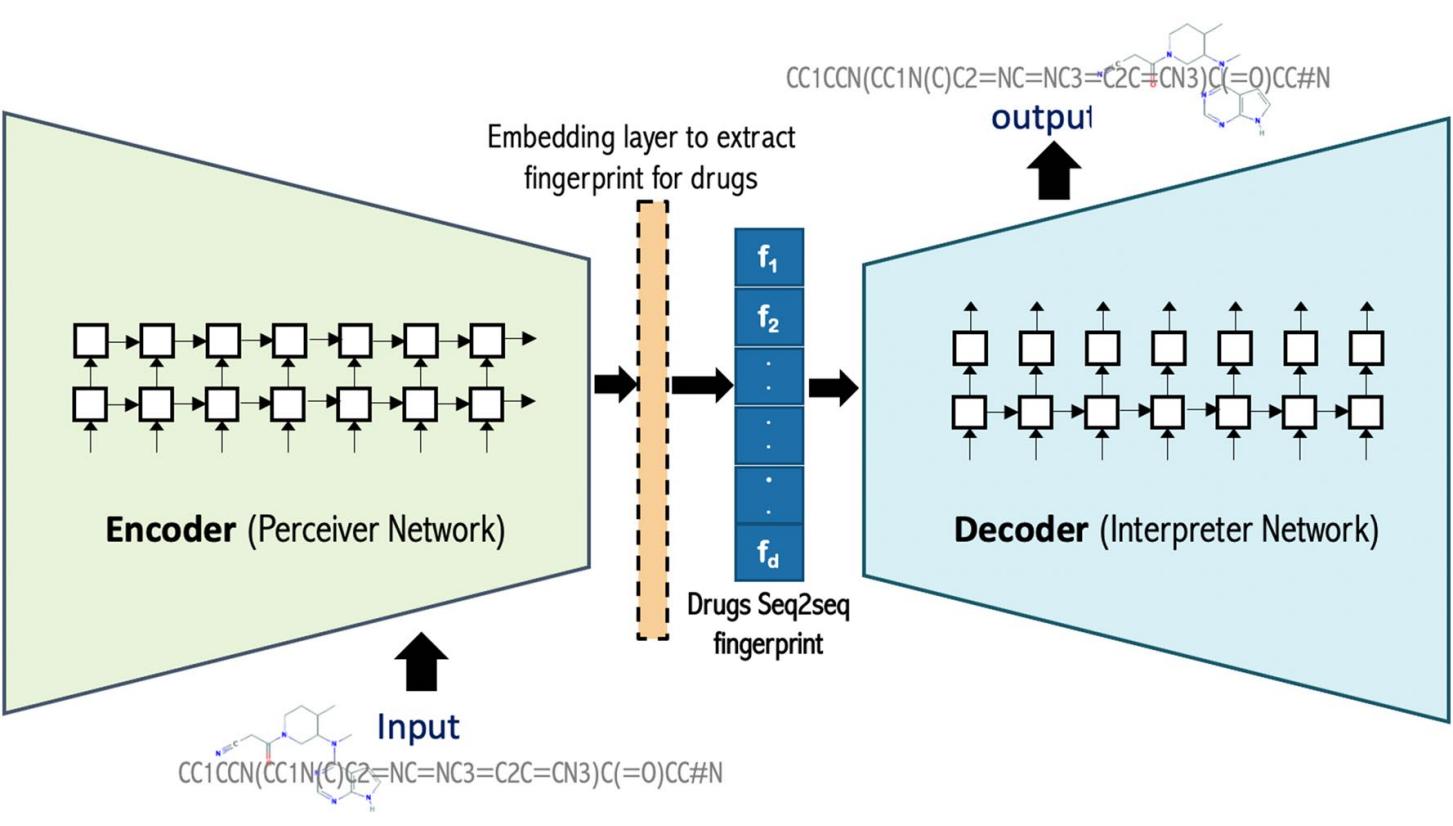
The seq2seq fingerprint architecture used to generate drug embeddings. For the drug’s SMILES 2D structure image we used the smi2img tool http://hulab.rxnfinder.org/smi2img/.

Figure 2 shows the overview of the seq2seq model that we utilized and then applied. The input and the output of the seq2seq model are the SMILES strings that serve as the text representation. That is, we fed the drugs’ SMILES to the model so that the perceiver converts the SMILES strings into a fixed-sized FV, and then the interpreter translates it back to the original SMILES strings. The intermediate fixed-sized FV is extracted as the seq2seq fingerprint. The intermediate FV encodes all the essential information to recover the original drugs' representation. Hence, we expect the seq2seq fingerprint to capture the precise information we can use for downstream ML tasks. In our work, we trained the model using around 85,900 SMILES, which we split into the tokens that can appear in the SMILES string (i.e., the alphabet for the seq2seq model). We assigned these tokens with the maximum SMILES length to the seq2seq model with other parameters. Furthermore, we utilized several parameters of our seq2seq model using different sets of tested values (see Table 2), and we applied Adam optimizer in the DL training process. Finally, we extracted the embeddings for each drug in Davis, KIBA, and PDBBind Refined datasets by predicting each drug's SMILES features.

**Table 2:** The seq2seq model’s parameter optimization, bold fonts indicate the selected values.

Parameters	Tested values
Feature Vector length	{**128, 256**, 512, 1024}
Learning rate	{0.1, **0.01**, 0.001}
Number of layers	{**2**, 3, 4}
Batch size	{5, **10, 100**}
Dropout value	{0, **0.1**, 0.2, 0.3}
Variational autoencoder	(True, **False**)

### Protein encoder

**Figure 3:**
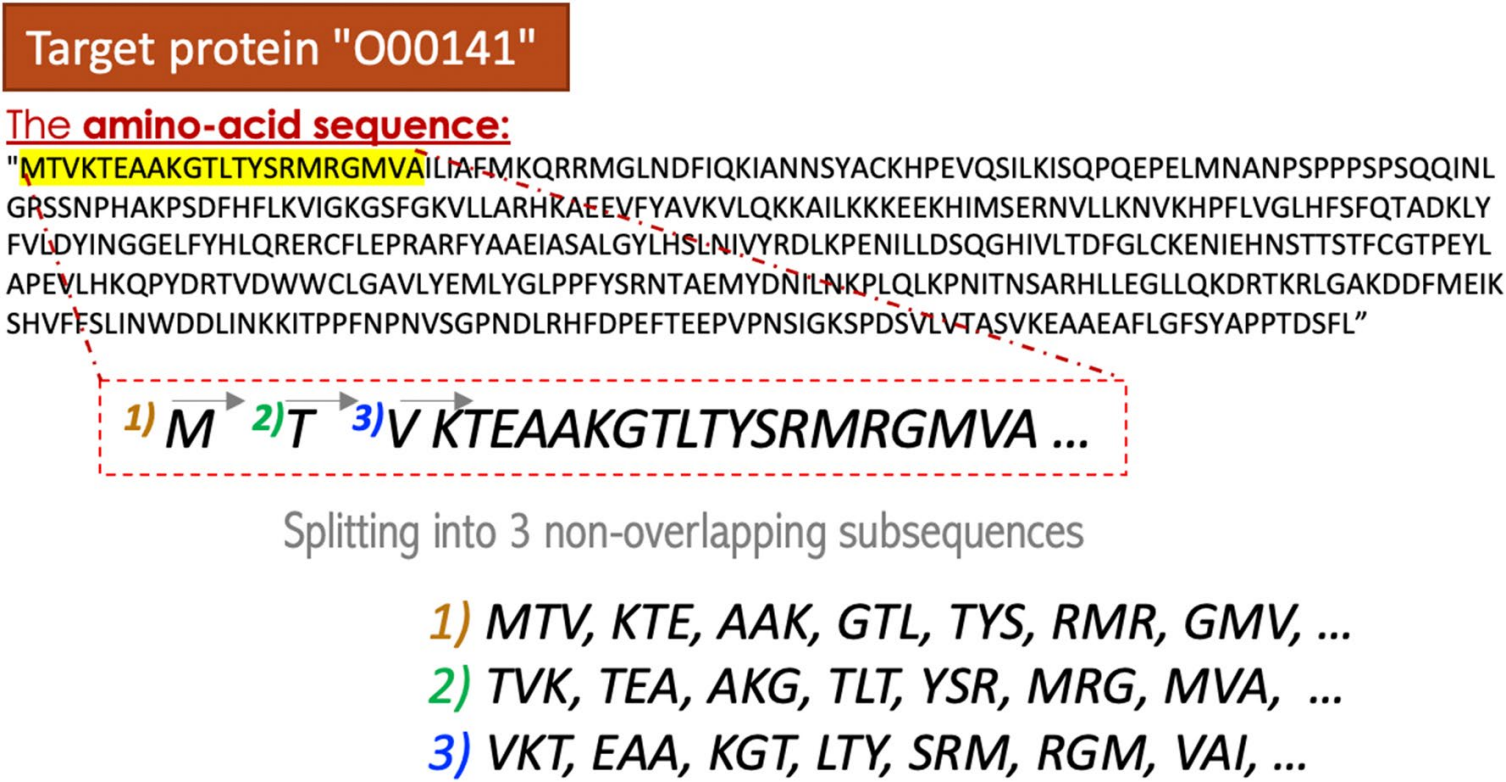
The process used to generate three lists of non-overlapping biological words that served as the corpus for ProtVec training.

To generate meaningful feature representations for the target proteins, we applied ProtVec^53^, a feature extraction method for protein amino-acid sequences. ProtVec^53^ is an unsupervised data-driven continuous distributed representation of biological sequences that captures a diverse range of informative biophysical and biochemical characteristics. Similar to the Skip-gram word embeddings model's training process in natural language processing (NLP), a large corpus is needed to train distributed representation of biological sequences. Thus in this work, for ProtVec^53^, 546,790 amino-acid sequences of different proteins were downloaded from the SwissProt UniProt server^54^. Then, those sequences were broken down into subsequences (i.e., biological words) using the n-gram model. However, instead of using an overlapping window of 3 to 6 residues, they generated three lists of shifted non-overlapping words (*3-gram is a "biological" word consisting of 3 amino acids*), as shown in Fig. 3. Thus, the ProtVec is trained based on 1,640,370 (546,790 × 3) sequences of 3-gram through a Skip-gram neural network model that maximize the probability of observed word sequences. We used the pre-trained model to generate the embeddings for each target protein in our datasets. Then we represent each protein sequence by summing up the overlapping 3-gram feature representation.

### Feature extraction model

We developed three ways to extract the features for each drug-target pair:

1. We applied graph mining techniques to compute the drug-target meta-path score features.
2. We used the embedding FV for each drug and target described in the previous section.
3. We produced a hybrid model that combines the features from the first two ways.

These features capture the characteristics of the target proteins, drugs, and DTBA network.

### Meta-path-based features

After learning feature representation for each target and drug, we calculated the cosine similarity (CosSim) between two drugs vector representations (i.e., *drug*_*i*_ and *drug*_*j*_ embeddings corresponding to *v*_*i*_ and *v*_*j,*_ respectively) for each pair of drugs as follows:

4 $$CosSim({v}_{i},{v}_{j})=({v}_{i}. {v}_{j}) /( \Vert {v}_{i}\Vert . \Vert {v}_{j}\Vert )$$

Likewise, we applied the same process to target proteins. Therefore, we constructed two new similarity matrices, which are *TTsim2*, target-target similarity matrix with size m*m, where m is the number of targets, and *DDsim2*, drug-drug similarity matrix of size n*n where n is the number of drugs. Then, we applied min–max normalization on each similarity graph to have all similarity scores between 0 and 1. At this stage, we created two different extensive weighted heterogeneous networks DTBA with the same binding affinity graph but with different drug-drug and target-target similarity graphs: ***G1****(DTBA, TTsim1* subgraph*, DDsim1* subgraph*)*, and ***G2****(DTBA, TTsim2* graph*, DDsim2* graph*)*. It is necessary to state that we excluded all test data in our model's training process, including the binding affinity edges that belong to the test set. Then, we applied graph mining techniques similar to^55^ to extract features from these two graphs *G1* and *G2*, and then either used the features from each graph individually or combined them. For each weighted heterogeneous graphs *G1* and *G2*, we computed each drug-target path scores as described in Eq. (5):

5 $$score\left({d}_{i},{t}_{j}\right)= \sum_{p=1}^{n}\prod {P}_{weights}$$

*P* = {*p*_*1*_*, p*_*2*_*,…, p*_*q*_*, …, p*_*l*_} is the set of paths from drug_i_ to target_j_. In our work, we limited the path length to be equal to or less than three because of computational complexity. Thus, we obtained six path structures. Each path structure starts with a drug node and ends with a target node, and there is no cycle in the path (each node in the path appears only once). These path structures are represented by C_h_ where *h* = {*1, 2, 3, 4, 5, 6*}, which are: (C_1_: (D-D-T), C_2_: (D-T-T), C_3_: (D-D-D-T), C_4_: (D-T-T-T), C_5_: (D-D-T-T), and C_6_: (D-T-D-T). The meta-path score for each path *P*_*q*_ is the product of all weights *w*_*x*_ of edges from *d*_*i*_ (drug node) to *t*_*j*_ (target node) in each path structure belongs to C_h_, as follows:

6 $$score\left(h,{d}_{i}, {t}_{j}, q\right)=\prod_{{\forall w}_{x}\in {P}_{q}, { P}_{q}\in {R}_{ijh}}\left({w}_{x}\right)$$

The weight of similarity edges between two nodes of the same type indicates the transition probability between those nodes, and the weight of the preprocessed values of binding affinity edges indicate the probable strength with which drugs bind to the target proteins (the larger probability indicates a more significant degree of binding affinity). Therefore, multiplying edge weights ranging between [0,1] across the path penalizes longer paths, which fits our objective, contrary to the summing of edge weights across longer paths, which conflicts with our goal. Following our previous published work^55^, we obtained 12 meta-path-based scores by calculating the sum and max scores (i.e., features) under each path structure as shown in Eqs. (7) and (8), respectively.

7 $$SumScore\left(h,{d}_{i}, {t}_{j}\right)= {\sum }_{\forall q: { P}_{q}\in {R}_{ijh}}score\left(h,{d}_{i}, {t}_{j},q\right)$$

8 $$MaxScore\left(h,{d}_{i}, {t}_{j}\right)= {MAX}_{\forall q: { P}_{q}\in {R}_{ijh}}\left(score\left(h,{d}_{i}, {t}_{j},q\right)\right)$$

where *R*_*ijh*_ is the set of all paths under all path structures between drug_i_ and target_j_. We applied the same procedure to both *G1* and *G2*, which means our FV dimension is either 12 or 24 when we combine *G1* and *G2* FVs. It is worth mentioning that the meta-path score features were encoded in commuting matrices as introduced in^56^, which we calculated by multiplying two or three adjacency matrices. The length of the meta-path equals the number of multiplied adjacency matrices which are: DTBA, target-target similarity, and drug-drug similarity. Using 3D matrix multiplication accelerates the running time to get the meta-path scores.

### Embedding-based features

After automatically generating the feature representations for each drug and each target using different DL techniques (seq2seq model for drugs and ProtVec model for proteins), we obtained two embedding matrices *f* and *Pv* for drugs, and targets, respectively. Drug embedding matrix *f* has the size equal to *|n| x d* (*n* is the number of drugs and *d* is the feature dimension)*,* and the target embedding matrix *Pv* has a size equal to *|m| x k* (*m* is the number of targets and *k* is the feature dimension which is 100). Then, because our goal is to predict drugs and targets potentially linked with their binding values, we created an embedding FV for each drug-target pair by applying the concatenation operation, as shown in Fig. 1c. Thus, for any possible drug-target pair, we concatenated a drug FV with target FV resulting in a longer FV which has the dimension of *d* + *k* (i.e., number of the features) and the number of the samples are *n x m* (i.e., number of drugs multiplied by the number of targets).

### Hybrid model-based features

In this step, we concatenated the meta-path scores features with the drug embedding features and target embedding features, for all drug-target pairs.

### Regression model

After extracting all the drug-target pairs' features, we normalized the features for the training and testing sets separately using min–max normalization^57^. Then, the normalized FVs were fed with their label Y (i.e., binding affinity values) into a supervised ML regression model to predict the binding affinity values. Any models’ prediction performance relies on identifying the most critical features of the studied dataset. Thus, we developed three models using different sets of features (explained above) and tuned the models' parameters using these feature sets. We named these models *Affinity2Vec_Pscore* (when we use the graph-based features), *Affinity2Vec_Embed* (when we use embedding-based features), and *Affinity2Vec_Hybrid* (when we combine both sets of features).

Unlike the well-known parametric linear regression model that assumes the target variable can be expressed as a linear combination of the independent variables, the gradient boosted trees are nonparametric models that approximate any distribution function from the data. They usually achieve better prediction performance than linear regressors. Hence, we utilized gradient boosting regression, particularly Extreme Gradient Boosting Regressor (XGBoost)^58^. XGBoost regressor is implemented using an optimized distributed gradient boosting library named XGBoost^59^ that is highly efficient, flexible, and portable. The objective of XGBoost in the prediction process is to minimize the loss function, which is, in our case, the mean square error (MSE).

We selected XGBoost because:

1. It is much faster than other ensemble regressors.
2. Its core algorithm is parallelizable, meaning that it exploits the power of the multi-core machine and works on GPUs.
3. It can be optimized during the validation process to enhance the performance because it has a wide variety of tuning parameters.

In this study, all the Affinity2Vec models utilized ML XGBoost regressor and DL regressor to predict the affinity values. The DL model that we utilized is a feedforward artificial neural network (ANN) that consists of three fully connected (FC) layers. For activation function, we used the rectified linear activation function (ReLU). XGBoost performed better than DL in our experiments and this result may be a consequence of DL models working better with larger feature numbers. Thus, we only reported the results of XGBoost. The most critical parameters that we optimized include but are not limited to: 1/ the number of trees in the ensemble, which is usually tuned by increasing the trees until we see no further improvements, 2/ the maximum depth of each tree, 3/ the learning rate (often set to small values such 0.1, 0.01), 4/ the subsample which is the number of the data samples used in each tree, and 5/ colsample_bytree which is the number of features used in each tree, set to a value of 1 when using all features.

### Evaluation metrics

To acquire a more realistic view of Affinity2Vec's prediction performance, we implemented the same evaluation metrics widely used to evaluate the regression-based state-of-the-art methods. These metrics are: the mean squared error (MSE)^60^, the concordance index (CI)^61^, the regression toward the mean index (rm2)^62^, and the area under the precision-recall curve (AUPR)^63^.

#### Mean square error (MSE)

MSE^60^ is the most popular metric to evaluate regression-based models to measure how close the fitted line is to the actual data points. Thus, MSE is used as a loss function that the training model tries to minimize. MSE is defined as follows:

9 $$MSE = 1/n {\sum }_{i=1}^{n }{({p}_{i }-{y}_{i} )}^{2}$$

where ***p*** represents the prediction values vector, y represents the actual values vector, n is the number of samples, and MSE is the average sum of the square difference between the predicted values and the actual values. The formula uses the square to ensure the negative values do not cancel the positive values. The smaller the value of MSE, the better the regression model's performance. Root mean squared error (RMSE) is the square root of MSE, used as a substitute for MSE in several studies.

#### Concordance index (CI)

CI has been widely used to evaluate regression models' ranking performance^61^. The CI of a set of paired data is equal to the probability that two random pairs with different label values are predicted in the correct order defines as follows:

10 $$CI = 1/ Z \sum_{dx>dy} h (bx - by)$$

*bx* and *by* are the prediction values for the larger affinity *dx* and the smaller affinity *dy*, respectively, *Z* is the normalization constant, and *h(m)* is the Heaviside step function^64^ defined as follows:

11 $$h\left(m\right)=\left\{\begin{array}{c}1, \quad if\, m>0\\ 0.5,\quad if\, m=0\\ 0,\quad if\, m<0\end{array}\right.$$

#### Regression toward the Mean (rm2 index)

The *rm2*^65^ has been extensively used to validate regression‐based quantitative structure–activity relationship (QSAR) models. *rm2* is a modified version of the squared correlation coefficient, also known as determination coefficient, (*r*^*2*^), implemented to evaluate the binding affinity model's external predictive potential. If *rm*2 is greater than 0.5 on the test data, it indicates the performance is acceptable. *rm*2 is defined using two other evaluation metrics: *r*^*2*^^66^ is the proportion of variation in the outcome described by the predictor variables. The *r*^*2*^ corresponds to the squared correlation between the actual values and the predicted values in multiple regression models, while ${r}_{0}^{2}$ is a squared correlation coefficient with zero intercepts:

12 $${r}_{m}^{2} = {r}^{2} \times \left(1 - \sqrt{{r}^{2} - {r}_{0}^{2}}\right)$$

More details of the formulation are explained in^65,66^. The higher the *r*^*2*^ and *rm*2, the better the performance of the regressor. Note, we also used *r*^*2*^ in a nonparametric statistical approach called *Y*-Randomization (also known as *Y*-Scrambling)^67^, that served as the final validation step.

#### Area under precision-recall curve (AUPR)

AUPR evaluation metric^63^ frequently used with binary classification problems known to perform well with imbalanced data since it differentiates the predicted scores of the positive data samples and the predicted scores of negative data samples. We utilized AUPR to evaluate our Affinity2Vec’s performance in binary classification prediction. The closer the value of AUPR is to 1, the better the performance. We can easily convert our datasets into their binary shapes by setting specific binding affinity thresholds for each dataset separately. Thus, if the binding affinity value is above the threshold, it is transformed to 1 (known interaction); otherwise, it will be zero. Our study follows the suggested thresholds from prior work^23,39,68^, where we set the threshold values to 7 and 12.1 for the Davis and KIBA datasets, respectively. For the PDBBind Refined dataset, we specified 6, 7, and 10 as the thresholds following the previous work^44^. We calculated the AUPR using each threshold and then calculated the average AUPR.

### Experimental settings

We applied two settings to evaluate our models. For setting-1, like the state-of-the-art methods, we utilized the nested cross-validation (CV) approach to evaluate Affinity2Vec’s. The advantage of the nested CV approach over the regular CV is that it attempts to overcome bias and overfit the training data. Thus, it tunes the hyperparameters of the regressors in the training stage. The nested CV procedure that we applied is as follows: we randomly partitioned the dataset into six equal sets, one of them used as a hold-out set for testing, and the other five sets for training fivefold CV. Hence, all four evaluation measurements obtained are for the five models' averages on the hold-out test data. In addition, as mentioned before, we eliminated all binding affinity edges that belong to the test data from the graph that we formulated. To make the performance comparison unbiased, as fair as possible, we used the same benchmark datasets (Davis and KIBA) and setting of nested CVs, applied the same splits of the training and testing data as the current state-of-the-art methods, and finally, utilized the same four evaluation metrics.

For settings-2, we followed the same setting process of prior works^42^, using: a time-based splitting protocol, the same dataset (PDBBind Refined v.2015), and the same evaluation metrics. Thus, we split the dataset into three subsets: training, validation, and test sets based on the publication years that were provided with the dataset. For training data, we used all protein–ligand complexes from the year 2011 and before. For the validation data, we used protein–ligand complexes for 2012, while for test data, we used all complexes from 2013 and after. Therefore, we obtained 2188, 312, and 547 data samples (ligand–protein complexes) for training, validation, and testing sets, respectively.

We executed all our experiments on a Linux Ubuntu 18.04.5 LTS Intel Xeon Platinum 8176 workstation, 64-bit OS, with 112 processors and 2 GPUs (Quadro and Titan) with CUDA version 11.0. For implementation, we used python 3.8 and some required libraries such as Keras^69^ for DL, DeepChem for seq2seq fingerprint model^51^, ProtVec^53^ for proteins’ embeddings, XGBoost^59^ for regression, and more.

## Results and discussion

Here we explain the results we obtained for the three benchmark datasets under two settings using the three models we developed. We further compared our model's performance, Affinity2Vec, with selected state-of-the-art methods and discussed our model's key strengths that might enhance our model performance.

### Affinity2Vec models’ performance

We conducted several experiments to predict DTBA and reported the best-obtained results among all variants of Affinity2Vec in MSE and CI for each model with the number of features that we used in each model using Davis and KIBA datasets in Table 3. We assessed three versions of Affinity2Vec models using distinct sets of FVs for each dataset separately. This process allowed us to test our methods' performance in multiple experiments and then select the model with the best-obtained results. Supplementary Table S1 provides the other evaluation metrics (rm2 and AUPR) for all the Affinity2Vec models.

**Table 3:** Best-obtained results among all variants of Affinity2Vec for Davis & KIBA datasets in term of MSE and CI.

Model name	Features type	Number of features	Davis Dataset		KIBA Dataset	
			MSE	CI	MSE	CI
Affinity2Vec Pscore	(a) Meta-path scores of G1	12	0.251	0.886	0.247	0.83
	(b) Meta-path scores of G2	12	0.35	0.85	**0.111**	**0.923**
	(c) Meta-path scores of G1 & G2	24	0.253	0.885	**0.121**	**0.91**
Affinity2Vec Embed	Dr SMILES embeddings FV + Pr aaseq embeddings FV	D (228) K (356)	0.339	0.857	0.295	0.80
Affinity2Vec Hybrid	(a) Meta-path scores of G1 + Dr SMILES embeddings FV + Pr aaseq embeddings FV	D (240) K (368)	**0.24**	**0.887**	0.194	0.854
	(b) Meta-path scores of G2 + Dr SMILES embeddings FV + Pr aaseq embeddings FV	D (240) K (368)	0.325	0.861	0.124	**0.91**
	(c) Meta-path scores of G1 & G2 + Dr SMILES embeddings FV + Pr aaseq embeddings FV	D (252) K (380)	**0.242**	**0.886**	0.124	0.905

Bold font with underline indicates the best results, and bold font alone shows the second-best results.

*Dr* Drugs, *Pr* proteins, *aaseq* amino-acid sequence, *D* Davis dataset, *K* KIBA dataset. *G1* consists of (DTBA training part, *DDsim1* 2D chemical structures similarity, *TTsim1* targets’ sequence alignment similarity using normalized Smith-waterman scores), G2 consists of (DTBA training part, *DDsim2* drugs’ SMILES embeddings cosine similarity, *TTsim2* targets’ sequence embeddings cosine similarity).

As shown in Table 3, we achieved the worst MSE and CI evaluation metrics for both datasets when only concatenating FVs of drugs' SMILES and targets' sequences embeddings and feeding them to the XGBoost model. However, we observe a slight improvement in prediction performance when we include embeddings in the hybrid models (compared to the Affinity2Vec_Pscore models). We further observe that Affinity2Vec_Pscore models perform better using both datasets. But the better results are achieved with *G2* that incorporate the embeddings in the meta-path score features in the form of cosine similarity for all drug pairs or all target pairs (specifically in the KIBA dataset). That demonstrates applying some process on the auto-generated features instead of concatenating them, making them more meaningful and informative, and contributing to the prediction positively. Furthermore, it shows the importance of formulating the DTBA as graph-based and graph mining power to obtain different meta-path scores.

The reason behind *G2* not performing well when using the Davis dataset may be a consequence of DL working better with larger datasets such as the KIBA dataset used in our work. This means both the seq2seq and ProtVec models capture the most significant features in the larger dataset (i.e., KIBA). Therefore, all models that used DL in some steps showed better performance in the KIBA dataset.

Moreover, the Affinity2Vec_Pscore model showed different performance for both datasets when we used the meta-path scores from *G1* or *G2*. The only difference between *G1* and *G2 *was the source of obtaining similarity graphs of drugs or for targets. Thus, the similarity types play an essential role in the graph-based features. Furthermore, the Davis dataset results show that using *DDsim1* and *TTsim1* (from the 2D chemical structure and normalized SW alignment scores for drugs and targets, respectively) enhances the performance when involved in the FVs in four versions of our model. In contrast, for the KIBA dataset, the *DDsim2* and *TTsim2* (from SMILES embeddings cosine similarity and amino-acid sequence embeddings cosine similarity) improved the performance when they were involved in the FVs. As a result, it is a fundamental and critical process to choose the most informative similarity to integrate with the DTBA weighted heterogeneous network to calculate path score features. Besides, we observe that the Affinity2Vec_Pscore model outperformed the other models' variants in the KIBA dataset. We can pick up the importance of the meta-path score features from the performance results, which show that the Affinity2Vec_Hybrid, which combines both types of features, is very similar to the Affinity2Vec_Pscore model. This is despite Affinity2Vec_Pscore only having 12/24 features and Affinity2Vec_Embed having 228/356 features. Thus, it seems the meta-path score features are dominating the results. This suggests graph-based features were constructed based on the popular "guilt-by-association" assumption, similar drugs tend to interact with similar targets and vise verse, can exploit the graph structure to provide more meaningful information that extends beyond the number of features used. This outstanding characteristic significantly improved the DTBA prediction performance in our work.

As a final step, we further validate our ML models' robustness and stability by demonstrating that the strong performance obtained was not by chance. That is, we implemented *Y*-Randomization^67^ to validate the quantitative structure/property-activity relationship (QSPR/QSAR). We performed this test by comparing our model (trained on the original dataset) to several versions of our model trained on randomly shuffled datasets. Briefly, for the Davis and KIBA datasets separately, we first trained our model using the original data (i.e., features and labels) and obtained the results. Second, for 100 iterations, we fixed the FV but shuffled the labels, then trained the model over the new features-label pairs and obtained their performances. We performed the evaluations using leave-out test data in terms of *r*^*2*^ (explained in “Evaluation metrics”), an evaluation metric commonly used to measure the goodness-of-fit^67^. With this data, we demonstrate that Affinity2Vec obtains statistically significant (P-values < 0.05) prediction performances in terms of *r*^*2*^ using the Davis dataset with P-value = 1.47e−18 (*r*^*2*^ = 0.71), and the KIBA dataset with P-value = 8.57e−20 (*r*^*2*^ = 0.78), compared to 100 randomized models for each dataset that achieved non-significant results (P-values >  = 0.1 and negative *r*^*2*^ values). Negative *r*^*2*^ indicates poor performance and that the model does not follow the data trend (i.e., chosen by chance). Obtaining very low P-values proves a dependency between the features and the labels, and our ML classifier exploited these correlations to obtain good results.

### Performance comparison under setting-1

For the comparison with the state-of-the-art methods, we considered the non-structure-based methods. These include the first ML works developed in this field of DTBA prediction, KronRLS^20^ and SimBoost^21^; and state-of-the-art DL methods, DeepDTA^23^, GANsDTA^68^, and DeepCDA^27^. It is important to mention that the results reported for the state-of-the-art methods were retrieved directly from the original publications except for KronRLS and SimBoost, which are informed by^23^ using the same settings for performance measurement and same datasets (Davis and KIBA). Furthermore, we only reported the best-performed version for each state-of-the-art method and did not include the results for all the versions.

**Figure 4:**
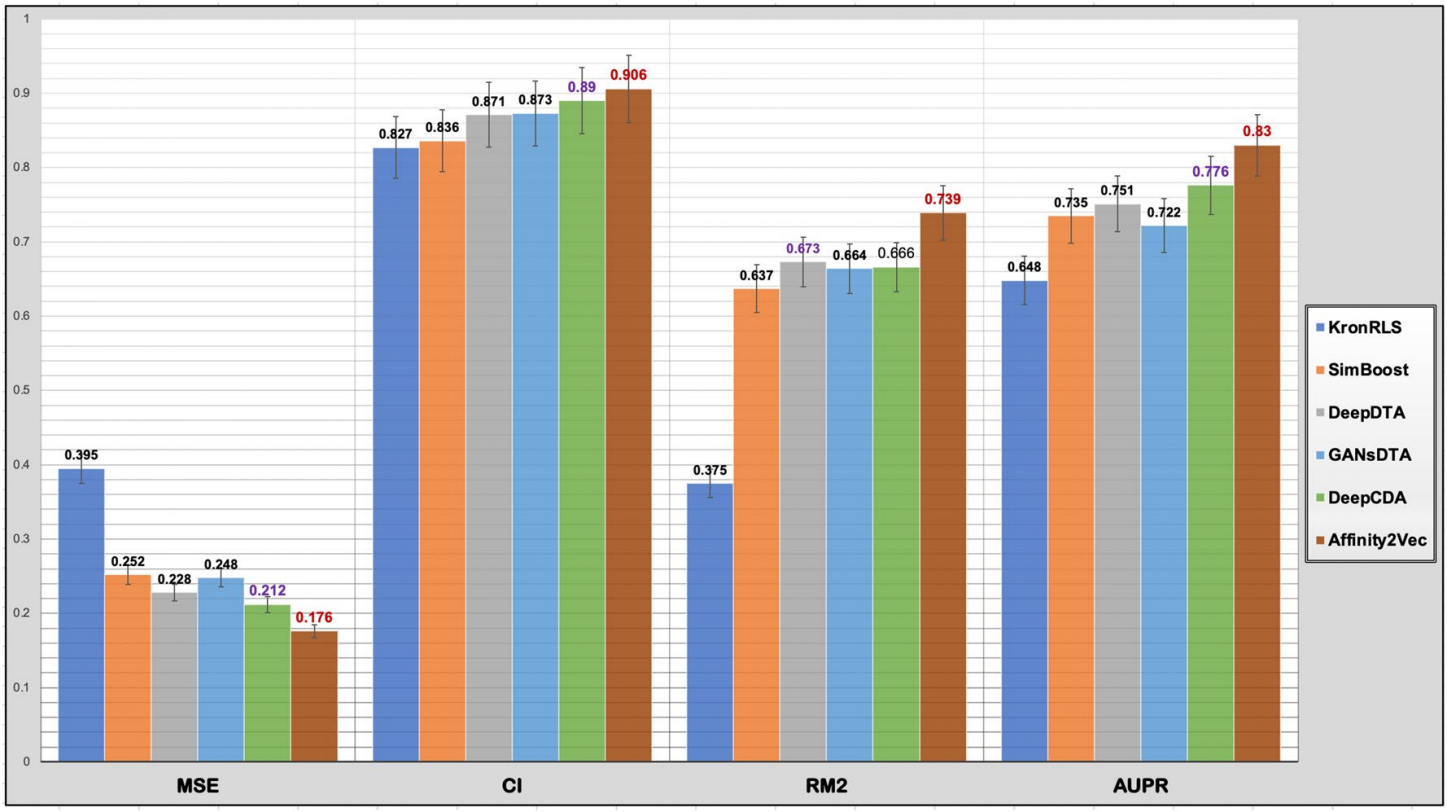
The average performance on the test set of each evaluation measurement (MSE, CI, rm2, and AUPR) across Davis and KIBA datasets for each method.

Figure 4 shows that Affinity2Vec (in brown) outperformed all the state-of-the-art methods by obtaining the best average in each evaluation metric across both datasets. Affinity2Vec achieved the highest average CI, rm2, and AUPR, which are around 1.6%, 6.6%, and 5.4% higher than the second-highest average CI, rm2, AUPR achieved, respectively. It also has the best average MSE across both datasets, which is 3.6% lower than the second-best method DeepCDA. This step (calculating the average for each evaluation metrics across all datasets) is vital because it provides a better assessment of overall method performance, which is independent of the dataset used.

Tables 4 and 5 show the average MSE, CI, rm2, AUPR evaluation metrics for our method and five state-of-the-art methods using Davis and KIBA dataset over fivefold CV on the hold-out test. Using the Davis dataset (see Table 4), our method, Affinity2Vec_Hybrid, achieved the best performance for two evaluation metrics, yielding the lowest MSE of 0.24 (considered the most important metric in regression-based problems) and the highest rm2 of 0.693. Affinity2Vec_Hybrid also is the second-best achieving competitive CI and AUPR. That is, its CI and AUPR are lower than the best-performing method (DeepCDA) in terms of CI and AUPR by 0.005 and 0.04, respectively.

**Table 4:** Comparing Affinity2Vec with five baseline methods in terms of CI, MSE, rm2, and AUPR scores for the Davis dataset on the test data.

Method	CI	MSE	rm^2^	AUPR	References
*KronRLS*	0.871	0.379	0.407	0.661	^20^
*SimBoost*	0.836	0.282	0.644	0.709	^21^
*DeepDTA*	0.878	0.261	*0.672*	0.714	^23^
*GANsDTA*	0.88	0.271	0.653	0.691	^68^
*DeepCDA*	**0.891**	**0.248**	0.649	**0.739**	^27^
*Affinity2Vec_Pscore*	*0.886*	*0.251*	**0.679**	*0.72*	
*Affinity2Vec_Hybrid*	**0.887**	**0.24**	**0.693**	**0.734**	

Bold font with underline indicates the best results, bold font alone shows the second-best results, and italic font shows the third-best results.

**Table 5:** Comparing Affinity2Vec with five baseline methods in terms of CI, MSE, rm^2^, and AUPR scores for the KIBA dataset on the test data.

Method	CI	MSE	rm2	AUPR	References
*KronRLS*	0.782	0.411	0.342	0.635	^20^
*SimBoost*	0.836	0.222	0.629	0.76	^21^,
*DeepDTA*	0.863	0.194	0.673	0.788	^23^
*GANsDTA*	0.866	0.224	0.675	0.753	^68^
*DeepCDA*	*0.889*	*0.176*	*0.682*	*0.812*	^27^
*Affinity2Vec_Pscore*	**0.923**	**0.111**	**0.783**	**0.926**	
*Affinity2Vec_Hybrid*	**0.91**	**0.124**	**0.765**	**0.91**	

Bold font with underline indicates the best results, bold font alone shows the second-best results, and italic font shows the third-best results.

Using the KIBA dataset (see Table 5), both Affinity2Vec_Pscore and Affinity2Vec_Hybrid, outperformed all five state-of-the-art methods in all evaluation metrics. Thus, we here compared the first ranked method to the third-ranked method. Specifically, the first rank version, Affinity2Vec_Pscore, improved the performance by 3.4%, 10%, and 11.4% for CI, rm2, and AUPR, respectively, compared to the second-best method (DeepCDA). It is noticeable that the CI score improvement is good, but both rm2 and AUPR scores have increased significantly compared with DeepCDA. Moreover, Affinity2Vec_Pscore proved its robustness by producing the lowest error, i.e., decreasing MSE to 0.112, a notable difference compared to the second-best method (DeepCDA), which is 6.5% higher.

Furthermore, we demonstrated the efficiency of our method by providing the top five drug-protein pairs based on the strongest binding (i.e., lower affinity values) predicted by the best Affinity2Vec model using the hold-out test set. Table 6 showed these drug-proteins pair along with their predicted and true affinities with other information. We can see that the predicted values are very close to the actual values, which indicates the robustness of our method. Moreover, in the KIBA dataset, for the first ranked predicted pair, the actual and predicted values are not very close, but it is the drug-target pair with the strongest actual binding affinity, and it is still recognized as such based on the predicted value, which reveals the robustness of our method.

**Table 6:** Predicted and actual affinity values of the top 5 drug-target pairs for Davis and KIBA datasets.

Dataset	Drug ID PubChem/CHEMBLE	Protein ID	Protein name (Primary gene name)	Predicted Aff values	Actual Aff values
Davis	3,025,986	P51451	Tyrosine-protein kinase Blk (BLK)	4.6658	5.00
	3,025,986	Q9H2G2	STE20-like serine/threonine-protein kinase (SLK)	4.6973	5.00
	10,138,260	P42680	Tyrosine-protein kinase Tec (TEC)	4.7683	5.00
	44,150,621	Q9UBE8	Serine/threonine-protein kinase (NLK)	4.7820	5.00
	17,755,052	O14976	Cyclin-G-associated kinase (GAK)	4.7956	5.5686
KIBA	CHEMBL373751	O14920	Inhibitor of nuclear factor kappa-B kinase subunit beta (IKBKB)	4.2933	6.09999
	CHEMBL281470	Q05655	Protein kinase C delta type (PRKCD)	9.0828	9.2397
	CHEMBL7929	Q05655	Protein kinase C delta type (PRKCD)	9.2187	9.4010
	CHEMBL2163772	P35968	Vascular endothelial growth factor receptor 2 (KDR)	9.2408	9.10003
	CHEMBL8163	P05771	Protein kinase C beta type (PRKCB)	9.3374	9.4010

The lower the affinity (AFF) value, the stronger the binding between drug and protein. The strongest binding affinity value in the Davis dataset is 5.00, while is 6.1 the strongest binding affinity value in the KIBA dataset after removing the zero values.

**Figure 5:**
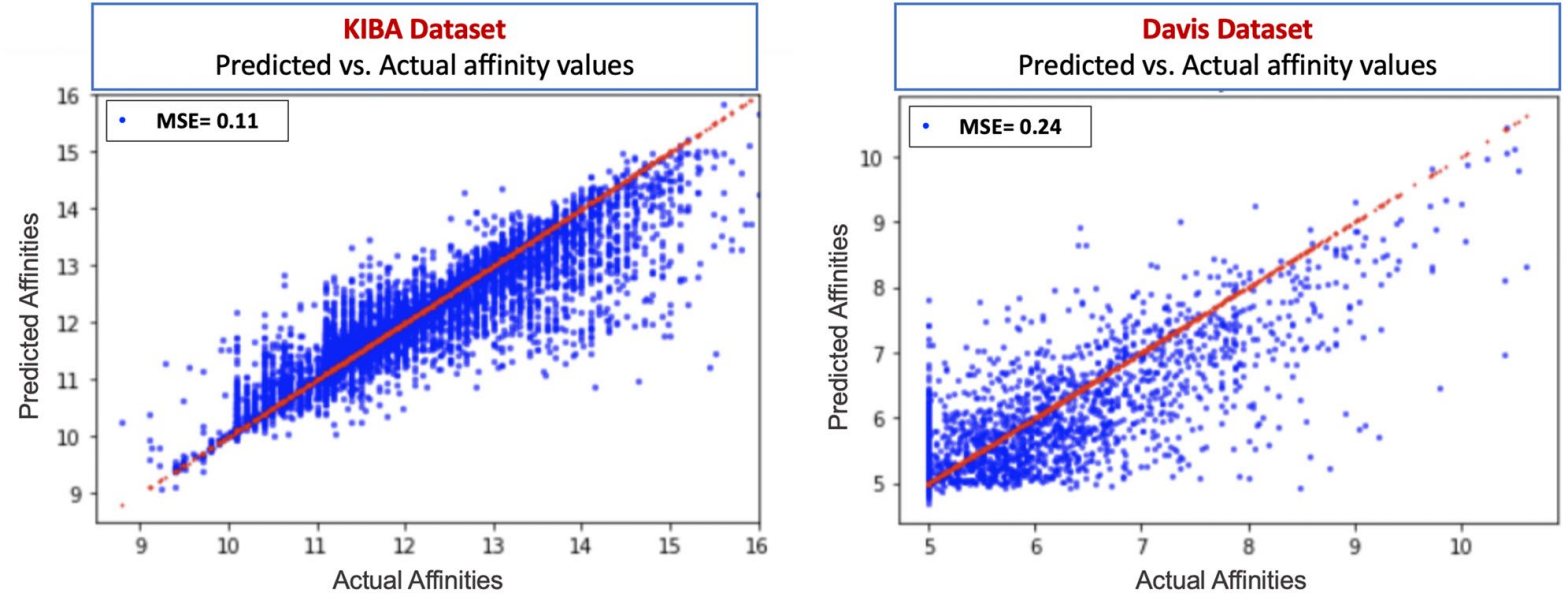
Binding affinities predicted by the Affinity2Vec best model vs. the actual binding affinity values for drug-target pairs in the Davis and KIBA datasets.

To further provide confidence in the predicted values, we also plot the predicted affinity measurements for the best Affinity2Vec model against the actual affinity measurements for both Davis and KIBA datasets using the hold-out test set. Figure 5 illustrates our claim by showing how close the predicted values (in blue) are to the actual values (in red). We expect the ideal predictive model to give the predictions (p) = actual values (y) (i.e., prediction on or close to the *x* = *y* line). The results suggest our model performs much better on the KIBA dataset, as we can see that the density is high around the *x* = *y* line.

### Performance evaluation and comparison using PDBBind dataset under setting-2

In this section, we assessed our methods’ performance in predicting binding affinity measurements using the benchmark PDBBind Refined dataset. Although the PDBBind Refined dataset provides the 3D structural information, we excluded this information to demonstrate that our method offers reasonable predictions based on this experimentally validated dataset despite not using the 3D data (thereby eliminating the bottleneck issue associated with the 3D structures). For a comprehensive evaluation, we assessed all three Affinity2Vec (Pscore, Embed, and Hybrid) models using the PDBBind Refined dataset under setting-2 (i.e., time-based dataset split) and compared them with two baseline methods that used the same dataset and the same setting. It is important to note that we have one Affinity2Vec model under each method version since using the PDBBind Refined dataset, we just obtained one graph G(V, E) based on the Ligands' SMILES embeddings cosine similarity and proteins' sequence embeddings cosine similarity.

Using the PDBBind Refined dataset and setting-2, we compared Affinity2Vec's performance with state-of-the-art methods, which applied the same experiment setup called MoleculeNet^7^, a benchmarking platform designed to evaluate computational methods for multiple molecular tasks, and MDeePred^8^, a multi-channel protein featurization DL-based method. We included four comparison methods from MoleculeNet since it utilized random forest (RF) and Deep Neural Networks (DNN) with grid featurization (GridF) and extended connectivity fingerprints (ECFP). The performance results of our models and these baseline models in terms of RMSE, CI, Spearman, and AUPR were based on the independent test set and are shown in Table 7. As we see here and from Table 7, we compared Affinity2Vec with baseline methods using the PDBBind dataset differing from the baseline methods (KronRLS, SimBoost, DeepDTA, GANsDTA, and DeepCDA) we compared with using Davis and KIBA dataset. All latter methods require several pre-calculations to train the models that were not available for the PDBBind Refined dataset. Furthermore, the predicted and the actual affinity values for the MoleculeNet methods were only available for the PDBBind Refined dataset. Therefore, all reported results for these baseline methods using PDBBind Refined dataset in this setting were taken from the previous work^8^.

**Table 7:** Performance results of Affinity2Vec, MDeePred, and the MoleculeNet benchmarking methods (under setting-2) based on the PDBBind refined dataset.

Method		RMSE	CI	Average AUPR
MDeePred		*1.591*	**0.754**	*0.768*
MoleculeNet	GridF-RF	1.844	**0.729**	0.723
	GridF-DNN	1.901	0.67	0.643
	ECFP-RF	1.791	0.657	0.638
	ECFP-DNN	2.292	0.608	0.545
Affinity2Vec	Embed model FV	**1.50**	*0.727*	**0.791**
	Pscore model FV	*1.76*	0.663	0.673
	Hybrid model FV	**1.512**	0.723	**0.784**

Bold font with underline (indicate the best results), bold font (indicate the second-best results), and italics (indicate the third-best results).

The results in Table 7 show that our methods performed well using the independent PDBBind Refined test sets under setting-2 for all our method versions. Among our methods, the Affinity2Vec_Embed model performed the best in all evaluation metrics RMSE, CI, and average AUPR. In contrast, the Affinity2Vec_Pscore model performed the worst, and the reason behind that might be because the graph we used to extract meta-path scores is very sparse due to the limited number of available affinity values in the PDBBind Refined dataset compared to the Davis and KIBA datasets. Moreover, Affinity2Vec offered the best and the second-best performances compared to the state-of-the-art methods by lowering the RMSE by 9.1% compared to the third-best (MDeePred method). Moreover, Affinity2Vec achieved competitive results in terms of CI by obtaining the third place lower than the second-best (MoleculeNet Grid-RF) by 0.002. Finally, using average AUPR, Affinity2Vec Embed and Hybrid models achieved the best and second-best, which indicates that our methods work well as classifiers after converting the continuous output to binary output.

In summary, based on all the reported results, Affinity2Vec showed superior performance using the larger dataset, KIBA, by outperforming all state-of-the-art methods based on all evaluation metrics, indicating that Affinity2Vec works better with large-scale biomedical data. But Affinity2Vec also performed on par with the best performing models when a smaller dataset like Davis is used. Furthermore, Affinity2Vecs’ performance was competitive using the PDBBind Refined dataset against the state-of-the-art methods (under a time-based split setting) where the test data was unseen during the training stage. Also, Affinity2Vec reported steady performance using various affinity thresholds in terms of average AUPR.

### Key strength of Affinity2Vec

The reasons behind Affinity2Vec outperforming several of the state-of-the-art methods are manifold. Firstly, some methods deal with the drug itself as a molecule graph but do not formulate the entire problem as graph-based or construct a heterogeneous graph using several data resources. Thus, as far as we are aware, this is the first network-based approach that predicts binding affinity, and no published works deal with the problem in a graph-based manner. It is essential to mention that most of the DL baseline methods used different DL representation learning techniques to auto-generate features (i.e., embeddings) for drugs and target proteins, as we also did. However, the different steps that we did gave our method significant advantages over baseline DL methods. These advantages are:

- We exploited the power of well-designed DL models developed in the biomedical domain specifically for drug embeddings or target embeddings by utilizing them to fit our goal for generating high-quality feature representations that help enhance the prediction performance.
- The postprocessing of auto-generated features gives them more meaning and more reliability. We post-processed these features by calculating the cosine similarity of drugs' embeddings and targets' embeddings and then incorporating those similarities in formulating the new weighted heterogeneous graph. Integrating these new similarities in the graph provides more information for extracting meta-path score features after removing all low score edges by setting cutoff thresholds. This whole process is the distinct characteristic in our work that extraordinarily improved the performance.

## Conclusion

It is more informative to predict the strength with which a drug binds to a target rather than just indicating the binary relation between the drug and its target. Thus, we developed Affinity2Vec, a novel network-based method to identify DTBA as a regression-based problem. We first built a weighted heterogeneous network by integrating: DTBA graph, target-target similarity graph, and drug-drug similarity graph. We applied several preprocessing steps on those graphs before the integration step. Our method, Affinity2Vec, exerted several techniques from representation learning (i.e., embedding), DL, graph mining, and ML. DL was used in the seq2seq model and the ProtVec n-gram model to automatically generate feature representations for drugs and target proteins, respectively. We applied graph mining to obtain meta-path score features, and then we fed the FVs to the ML regression model (specifically ensemble learning) for prediction. We performed a comprehensive evaluation using three benchmark datasets (Davis, KIBA, and PDBBind Refined) under two different settings, and Affinity2Vec showed consistent performance compared to several state-of-the-art methods and illustrated its robustness.

Despite our method's accurate prediction and high performance, Affinity2Vec still suffers from some weaknesses limiting its capability and optimal performance. It is worth mentioning that our method, Affinity2Vec, is limited to predict random drug-target pairs. Therefore, it cannot identify the interaction of new targets or for new drugs, and we intend to handle this limitation in future work. Also, we tested Affinity2Vec using three benchmark datasets, and it is crucial to apply our method on more large-scale datasets that are closer to actual scenarios and fit more to our objective of drug repositioning. Last, we represented the drugs as sequences (SMILES) instead of using the molecules graph representation, which is more informative.

For future work, we will work to enhance the prediction performance by utilizing different embedding techniques (i.e., graph embedding^70^, knowledge graph embedding^71^, and sequence embedding^72^). We will explore our model's interpretability, which gives us the strength to highlight the most critical features that we obtain from DL methods. We intend to apply Affinity2Vec to a case study of real-life associated with drug repurposing and then verify our model's selected predictions to confirm our results' clinical relevance experimentally. The last notable point is that our network-based method can be a generic solution to any similar problem formulated as a graph-based problem in biomedical domains such as drug-disease interaction networks and protein–protein interaction networks.

## Supplementary Information

Supplementary Information.

## Data Availability

The source of the Davis dataset is publicly available in (*accessed by May 2020*): http://staff.cs.utu.fi/~aatapa/data/DrugTarget/. Based on a previous published study, the source of the KIBA dataset is publicly available in: https://github.com/hkmztrk/DeepDTA/tree/master/data/kiba. The source of the PDBBind Refined dataset can be found in *(accessed by September 2021).* http://www.pdbbind.org.cn, *(Only need a registration to download the data), and in* https://github.com/cansyl/MDeePred/tree/master/training_files/PDBBind_Refined. The source code of the **Affinity2Vec methods** and all three datasets are available on a GitHub repository at: https://github.com/MahaThafar/Affinity2Vec.

## Abbreviations

DTIs: Drug-target interactions
DTBA: Drug-target binding affinity
ML: Machine learning
DL: Deep learning
SW: Smith-Waterman
KIBA: Kinase Inhibitor BioActivity
SMILES: Simplified Molecular-Input Line-Entry system
aaseq: Amino-acid sequence
3D: Three-dimensional
NN: Neural network
NLP: Natural language processing
LSTM: Long-short-term memory
GRU: Gated Recurrent Unit
FV: Feature vectors
TTsim: Target-target similarity matrix
DDsim: Drug-drug similarity matrix
DNN: Deep neural network
CNN: Convolutional neural network
seq2seq: Sequence-to-sequence
ANN: Artificial neural network
ReLU: Rectified linear activation
FC: Fully connected
XGBoost: EXtreme Gradient Boosting
MSE: Mean square error
CI: Concordance index
rm2: Regression toward the mean index
AUPR: The area under the precision-recall curve
CV: Cross validation
