# Supplementary material for: Affinity2Vec: drug-target binding affinity prediction through representation learning, graph mining, and machine learning
Source: Sci Rep. 2022 Mar 19;12:4751. doi: 10.1038/s41598-022-08787-9 (PMC8934358; doi:10.1038/s41598-022-08787-9)
Supplement: Supplementary file 1 — Supplementary Information. [file 41598_2022_8787_MOESM1_ESM.pdf]

Affinity2Vec: Drug-Target Binding Affinity Prediction through Representation Learning, Graph Mining, and Machine Learning

SUPPLEMENTARY MATERIAL

Maha A. Thafar[1,2], Mona Alshahrani[3], Somayah Albaradei[4], Takashi Gojobori[1], Magbubah Essack[1*], Xin Gao[1*]

*To whom correspondence should be addressed.

Supplementary Figure with explanation

Supplementary Figure S1: An illustration of setting the thresholds for drug similarity and target similarity for the Davis dataset.
We did an empirical analysis for the drug-drug similarity graph and target-target similarity graph to specify different threshold values for each graph separately, as explained and illustrated in supplementary figure S1 in the supplementary material file. In particular, for the Davis dataset as an example, the threshold for drug similarity is set to 0.3 while the threshold of target similarity is set to 0.04. We plotted all occurrences of similarity below several thresholds ranging from 0.02 to 0.9 as shown in the figure below, and we assign the threshold value when there is a big changing in the curve.

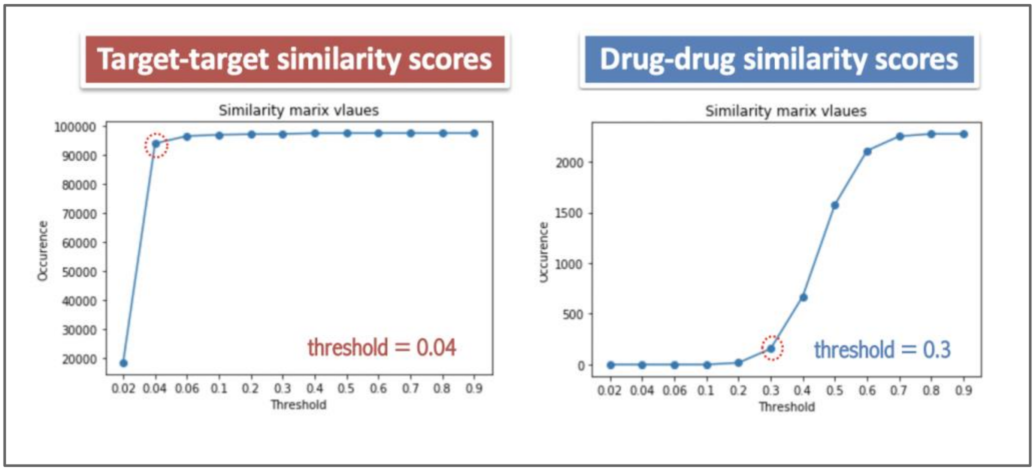

Supplementary Table

Supplementary Table S1: Best-obtained performance of several Affinity2Vec models using different features for Davis & KIBA Datasets in term of rm2 and AUPR - <u>Bold font with underline indicates the best results, and bold font alone shows the second-best results</u>.
The abbreviation in the tables: Dr: Drugs, Pr: Proteins, aaseq: amino-acid sequence, D: Davis dataset, and K: KIBA dataset. G1 consists of (DTBA training part, DDsim1 2D chemical structures similarity, TTsim1: targets' sequence

alignment similarity using normalized Smith-waterman scores), G2 consists of (DTBA training part, DDsim2 drugs' SMILES embeddings cosine similarity, TTsim2: targets' sequence embeddings cosine similarity)

Model name	Features Type	Davis dataset		KIBA dataset	
		rm2	AUPR	rm2	AUPR
**Affinity2Vec Pscore**	Meta-path scores of G1	0.679	0.72	0.615	0.746
	Meta-path scores of G2	0.53	0.61	**0.783**	**0.926**
	Meta-path scores of G1 & G2	0.677	0.718	**0.767**	**0.909**
**Affinity2Vec Embed**	Dr SMILES embeddings FV + Pr aaseq embeddings FV	0.57	0.633	0.58	0.68
**Affinity2Vec Hybrid**	Meta-path scores of G1 + Dr SMILES embeddings FV + Pr aaseq embeddings FV	**0.693**	**0.734**	0.686	0.80
	Meta-path scores of G2 + Dr SMILES embeddings FV + Pr aaseq embeddings FV	0.58	0.64	**0.765**	**0.91**
	Meta-path scores of G1 & G2+ Dr SMILES embeddings FV + Pr aaseq embeddings FV	**0.69**	**0.731**	**0.766**	**0.902**
